# Sphenochoanal polyps in children — a systematic review (1995‒2021)

**DOI:** 10.1016/j.bjorl.2022.02.006

**Published:** 2022-03-21

**Authors:** Krystian Tywoniuk, Karolina Haber, Józef Mierzwiński

**Affiliations:** a Children's Hospital of Bydgoszcz, Department of Otolaryngology, Audiology and Phoniatrics, Pediatric Auditory Implant Program. Ul. Chodkiewicza 44, 85-667 Bydgoszcz, Poland; b Nicolaus Copernicus University, Collegium Medicum. Department of Preventive Nursing. Ul. Łukasiewicza 1, 85-821 Bydgoszcz, Poland

**Keywords:** Nasal polyps, Choanal polyps, Child

## Abstract

**Objectives:**

Nasal polyps that originate from the sphenoid sinus and reach the nasopharynx are called sphenochoanal polyps. Reports on sphenochoanal polyps in children have thus far been limited only to case reports. This review aims to describe and summarize clinical presentation, diagnosis, management, surgical approaches to the sphenochoanal polyps with recurrence rate after surgery in pediatric patients reported in the literature.

**Methods:**

A systematic literature review was performed using PubMed, MEDLINE and Cochrane Library Databases for articles published prior to December 2021 to identify all studies reporting on pediatric patients with sphenochoanal polyps. Clinical presentation, management options, surgical approaches and outcomes of applied management were extracted from included studies.

**Results:**

9 articles provided data on 11 eligible patients with an age range 3 years and 8 months–16 years. The commonest symptoms included: nasal obstruction, nasal discharge, and headache respectively. All patients were subjected to surgical treatment. No recurrences after the endoscopic sphenoidotomy were reported.

**Conclusion:**

Sphenochoanal polyps should be kept in mind in the differential diagnosis of unilateral nasal cavity or paranasal sinuses masses. Misdiagnosis can result in recurrences in patients with sphenochoanal polyp, who can be mistakenly diagnosed with antrochoanal polyp and underwent inadequate treatment not involving sphenoidotomy and exact identification of the site of implantation. The symptoms of sphenochoanal polyps are nonspecific.

## Highlights

• Nasal polyps that originate from the sphenoid sinus and reach the nasopharynx are called sphenochoanal polyps.
• Only 11 cases of sphenochoanal polyps in children have been described in the world English-language literature.
• The symptoms of sphenochoanal polyps are nonspecific. The misdiagnosis can result in recurrences in patients with sphenochoanal polyp.
• Sphenochoanal polyps should be kept in mind in the differential diagnosis of unilateral nasal cavity or paranasal sinuses masses.

## Introduction

A choanal polyp is a benign solitary mass originating from the edematous and inflamed mucosa of the paranasal sinuses, passing through the sinus ostium, located within the nasal cavity, and extending to the nasopharynx with a wide pedicle.^1^ Nasal choanal polyps occur in three different forms: sphenochoanal, antrochoanal, and ethmoidochoanal polyps.^2^ Out of these 3 pathologies antrochoanal polyps are the most frequently reported while sphenochoanal and ethmochoanal polyps are very rare.^1^

Antrochoanal polyp (ACP) is a maxillary sinus polyp that originates in the maxillary sinus, grows through the maxillary ostium into the nasal cavity, without bone erosion or expansion, and extends posteriorly towards the choana.^3^, ^4^ It represents around 10% of all nasal polyps in adults, and 33% in children.^5^

Sphenochoanal polyps are an uncommon form of choanal polyp.^2^ They originate from the sphenoid sinus wall, exit the sinus via the sphenoid ostium, pass through the sphenoethmoidal recess, and reach the choana.^2^ They have intrasinusoidal, ostial, and extra-sinusoidal components.^2^ They are even more rare than antrochoanal polyps. Therefore, sphenochoanal polyp can be mistaken for its more common counterpart — the antrochoanal polyp.^6^ In pediatric population the frequency has not been reported. Tosun et al. reported that almost 50% of sphenochoanal polyps reported in the literature occurred in children.^7^ The etiopathogenesis of antrochoanal and sphenochoanal polyps has still not been fully clarified.^8^

Similarily to antrochoanal polyps, the treatment should be always surgical. All parts of the polyp should be resected to avoid recurrence.^9^ Due to the proximity of the optic nerve and carotid artery they cannot be removed in uncontrolled way. Uncontrolled pulling on the nasal polyp with forceps is dangerous due to the proximity of the carotid artery and optic nerve, which can be damaged during surgery.^2^

With the development in the endoscopic surgery, these less invasive methods of treatment gained popularity in pediatric antrochoanal polyps’ surgery. The endoscopic sinus surgery is now considered the ‘gold standard’ — the first choice for primary treatment in children.^4^, ^10^ However, the recurrences of ACPs in children are still high.^10^

Reports on sphenochoanal polyps in children have thus far been limited to case reports. No systematic reviews on sphenochoanal polyps in children appeared up to date. Little attention has been paid to complex clinical presentation, diagnosis, management, surgical decision-making, and surgical approaches in children. As no larger studies exists, this review aims to describe and summarize clinical presentation, diagnosis, management, and surgical approaches to the sphenochoanal polyps in pediatric patients reported in the literature.

## Methods

**Figure 1:**
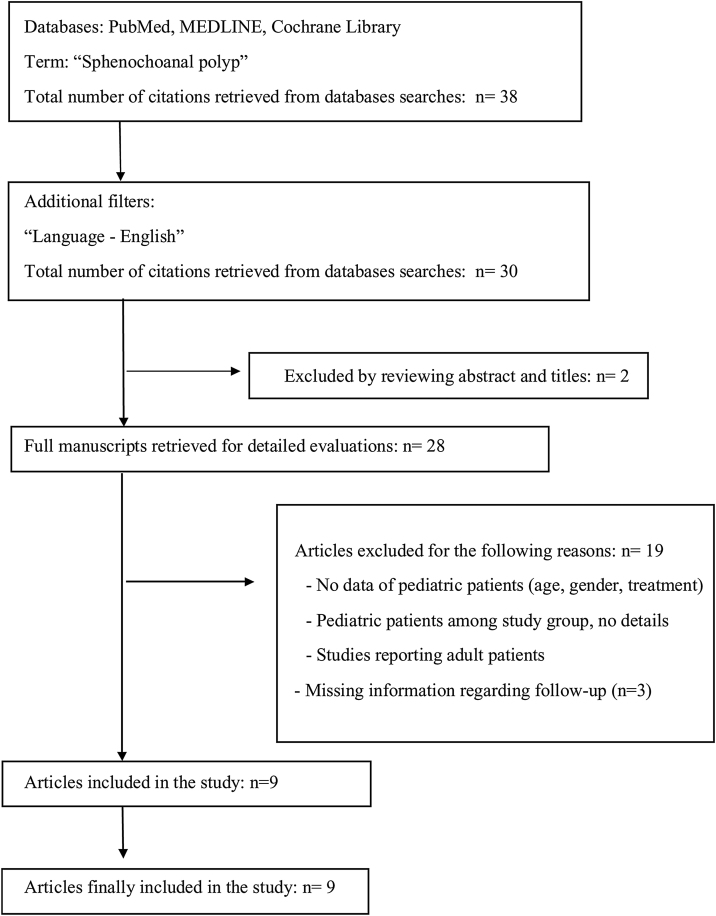
Search methodology.

A comprehensive literature search for all publications on sphenochoanal polyps in pediatric patients up to December 2021 was performed. The Preferred Reporting Items for Systematic Reviews and Meta-Analyses (PRISMA) guidelines were followed (Fig. 1).^11^ The last date for this search was 30th November 2021.

The Cochrane Library, MEDLINE as well as PubMed search engine (National Center for Biotechnology Information, Bethesda MD) were queried with the following search parameters: the term “sphenochoanal polyp”. The search query was further stratified by publication date in descending order. 38 publications were available; the list of publications was filtered by “Language — English”, reducing the total query to 30 publications. A two-researcher team then performed a systematic review of all 30 publications using the following inclusion criteria to select articles: (1) sphenochoanal polyp was the primary focus of the article; (2) sphenochoanal polyp was diagnosed in pediatric patient; (3) clinical presentation of the disease was described; (4) management of the sphenochoanal polyp was described; (5) follow-up period was described. If not available, the study was excluded. Data extraction from the articles included in the review was independently conducted by 2 reviewers and results were consistent.

Due to heterogeneity of the included studies, meta-analysis was not considered appropriate; therefore, only a qualitative analysis was conducted.

## Results

A diagram outlining the summary of the search process is shown in Fig. 1. After applying described filters, a total of 30 articles were identified. Screening by title and abstract excluded 2 articles. After screening the title and abstract, 28 full texts were reviewed. In total, of the 38 publications provided by primary search, 9 articles were identified between two reviewers as meeting the inclusion criteria, spanning from 1995 to 2019 (Fig. 1). All identified studies are shown in Table 1.

**Table 1:** Clinical presentation, diagnosis, management, surgical approaches to the sphenochoanal polyps with recurrence rate after surgery in pediatric patients reported in the literature.

Nº	Study	Number of children	Gender	Age (years)	Sympthoms	Side	Management	Reoperation	Complications	Follow up
1	Kumral et al.^2^	1	M	16	1-year history of bilateral nasal obstruction and discharge	Left	Endoscopic sinus surgery	No	While attempting to excise the polyp stalk, dehiscence in the course of the optic nerve was observed	Postoperatively, saline was applied for one week and antibiotic was given to the patient.
										The nasal cavity was free of polyps and the sphenoid sinus was clear radiologically

2	Çeçen et al.^1^	1	M	12	Nasal obstruction, olfactory dysfunction	Left	Endoscopic sphenoidotomy was performed.	No	None	No recurrence was seen in a three years endoscopic follow-up period
							The polyp was totally excised with its pedicle which was expanding the sphenoid sinus ostium together with the mucosa inside the sinus where the polyp originated.			
3	Özdek et al.^3^	1	F	15	Left-sided nasal obstruction and retro-orbital headache.	Left	The left sphenoid sinus ostium was enlarged endoscopically, and the polyp was removed totally	No	None	No evidence of recurrence was observed during the most recent follow-up, which occurred 15-months postoperatively
4	Al-Qudah et al.^12^	1	F	15	Nasal obstruction for 2-years, snoring, nasal discharge	Left	Endoscopic sinus surgery	No	None	No evidence of recurrence in 14-months following the procedure
5	Lim et al.^13^	1	F	3y8m	18-month history of mild to moderate obstructive sleep apnea	Left	The patient underwent adenoidectomy. During the procedure polypoid mass was diagnosed. Endoscopic sinus surgery was undertaken 5-months after adenotonsillectomy, with the purpose of excluding fungal sinus infection. Both nasal cavities were normal, and no mass was seen.	No	None	She remained asymptomatic at follow-up 2 months after the second surgery
							Bilateral transethmoid sphenoidotomy revealed normal sinuses			
6	Crampette et al.^14^	2	M	11	Nasal obstruction, infraorbital pain, snoring, nasal discharge	Right;	Endoscopic sinus surgery	Yes — patient underwent 2 surgeries	None	Four months later, the patient presented with similar symptoms, and a recurrence of the polyp at the same location was observed. After the second surgery and three-year follow-up no recurrence was observed.
							1st surgery: middle meatal antrostomy			
							2nd surgery: endoscopic sphenoidotomy			
			M	7	Nasal obstruction, snoring, headache, hearing loss	Right	Endoscopic sinus surgery	No	None	At six-month follow-up no recurrence was observed.
7	Íleri et al.^15^	1	F	14	Bilateral nasal obstruction for 11/2 years, snoring, nasal discharge	Left	Removal of the nasal lesion was performed under local anesthesia, the posterior part of the left middle turbinate was resected under endoscopic guidance, sphenoid sinus ostium, was widened.	No	None	No recurrence1 year postoperatively
8	Yanagisawa et al.^16^	1	F	14	Nasal congestion	Left	Resection of the sphenochoanal polyp began with amputation of the choanal portion of the polyp with a microdebrider. The sphenoid sinus ostium was enlarged with a microdebrider and the polyp was completely removed.	No	None	One year after surgery, the patient remains symptom-free and without evidence of recurrence
9	Tosun et al.^7^	2	M	15	Nasal obstruction, rhinorrhea, and headache for 1 year	Right	The patient was operated under local anesthesia.	No	None	There was no recurrence and no complications during 28-months of follow-up
							Nasopharyngeal part of the polyp was delivered through the mouth following disconnection from the pedicle			
							The sphenoid sinus ostium was widened, and the cystic part of the mass was completely taken out from the sphenoid sinus under endoscopic guidance			
			F	14	Nasal obstruction, headache and snoring for 2-years	Right	The patient was operated under local anesthesia.	No	None	The patient was symptom free and there was no complication or recurrence in the 18-months follow-up period.
							Following disconnection from pedicle, nasopharyngeal part of the polyp was taken out from the mouth. After widening the ostium, the pedicle, and the cystic part of the polyp in the sphenoid sinus were totally excised under endoscopic vision			

All studies combined encompassed 11 patients aged 18 years or younger. The overall mean age at diagnosis was 12 years with a range 3 years and 8 months–16 years. The male to female ratio was 4:7. 7 females and 4 males were included.

### Clinical presentation

The commonest reported symptoms included: nasal obstruction, nasal discharge, snoring and headache. In 10/11 patients’ nasal obstruction was primary symptom. 1 patient ‒ the youngest reported patient with sphenochoanal polyp – 3-year-old girl presented with 18-month history of mild to moderate obstructive sleep apnea as her only symptom.

All reported symptoms are shown in Table 2.

**Table 2:** Symptoms reported by pediatric patient with sphenochoanal polyps.

Presenting symptom/signs	Number (incidence)
Nasal obstruction	10/11 (90%)
Nasal discharge	5/11 (45%)
Snoring	5/11 (45%)
Headache	4/11 (36%)
Sleep apnea	1/11 (9%)
Facial pain	1/11 (9%)
Hearing loss	1/11 (9%)
Olfactory dysfunction	1/11 (9%)

## Diagnosis

Paranasal sinus Computed Tomography (CT) was done in 11 out of 11 patients (100%). Computed tomography of the nose and paranasal sinuses confirmed in all cases the presence of a mass in the nasal cavity, which extended to the choana with opacification of the sphenoid sinus. Magnetic Resonance Imaging (MRI) was performed for further evaluation in 1 child to exclude an encephalocele.

### Management and surgical approaches

11 out of 11 patients (100%) underwent surgical treatment. 10 out of 11 patients were subjected to endoscopic sinus surgery. In 2 patients, prior to endoscopic sinus surgery, nasopharyngeal part of the polyp was delivered through the mouth following disconnection from the pedicle.^7^

One case of spontaneous regression of a sphenochoanal polyp in a 3-year-old girl was observed during the surgery — bilateral transethmoid sphenoidotomy revealed normal sinuses.^13^

### Recurrence rate

The follow up ranged from 3-months to 3-years (mean 16-months). No evidence of recurrence after endoscopic sphenoidotomy was observed during the last follow-up in all patients.

However, in the group of patients subjected to endoscopic sinus surgery 1 recurrence was noted due to misdiagnosis and inadequate treatment (middle meatal antrostomy) in 1 patient with sphenochoanal polyp. The antrochoanal polyp was primarily diagnosed in this 11-year-old boy.^14^ The polyp was removed, the maxillary sinus was explored through a middle meatal antrostomy, and the mucosa was removed, but the site of the origin of the polyp was not found in the maxillary sinus.^14^ The post-operative diagnosis was “antrochoanal polyp”. Four months later, the symptoms returned and a recurrence of the polyp at the same location was observed.^14^ The CT scan showed an opacity of the right sphenoethmoidal recess and right sphenoid sinus.^14^ The boy was subjected to second surgery, during which wide sphenoidotomy was performed.^14^ The site of the origin of the polyp was located in the inferolateral part of the right sphenoid sinus. After three-year follow-up no recurrence was observed.

## Discussion

Sphenochoanal polyps are an uncommon form of choanal polyp. The first report of this rare entity is attributed to Zuckerkandl (1892).^7^ They are usually solitary, but may occur with concomitant nasal polyps.^2^ A solitary polyp in the posterior part of the nasal passage can easily be diagnosed on anterior rhinoscopy but differentiation of the choanal polyps by only its appearance is impossible.^7^ Other atypical sites of origin for choanal polyps have been reported in the literature; these sites include the anterior ethmoid sinus, the nasal septum, and the inferior and middle turbinates.^3^

The symptoms of sphenochoanal polyps are nonspecific. Most nasal polyps produce the same symptoms, which can include nasal obstruction, nasal discharge, headache, snoring, nose bleeds, and ear fullness associated with eustachian tube obstruction.^2^ Children most commonly present with nasal obstruction and discharge. On the other hand, nasal symptoms are less common in children with acute isolated sphenoid sinusitis. According to recent systematic review on acute isolated sphenoid sinusitis in children 98.6% patients with this entity presented with the primary symptom of a headache, 50.7% had a fever, and only 22.5% presented with nasal symptoms including nasal discharge and congestion.^17^ 32.4% presented with headache in isolation.^17^

In pediatric population the exact frequency of sphenochoanal polyps has not been reported. In 2001 Tosun et al. noted that a few case reports addressing the resection of sphenochoanal polyps have appeared in the literature and almost fifty percent of the sphenochoanal polyps were reported in children.^7^ However, the incidence was based only on 3 reports.

During the review process we identified 12 articles describing sphenochoanal polyps in children. All studies combined encompassed 15 patients aged 18 years or younger. However, 3 articles were not included in the study.^18^, ^19^, ^20^ In the 30 studies matching the first exclusion criteria in which the age of patients with sphenochoanal polyp was determined, we identified 34 patients with this rare entity. Among them we identified 19 adults (56%) and 15 children (44%). Among patients diagnosed with sphenochoanal polyp reported in the literature 44% were children — almost fifty percent of the sphenochoanal polyps were reported in children.

Sphenochoanal polyps can be misdiagnosed as hypertrophied inferior turbinate, adenoid vegetation, pansinusitis, and nasal polyps.^2^ However, thirteen important neural and vascular structures are adjacent to the sphenoid sinus.^21^ Therefore, sinus pathology can result in serious sequelae if diagnosis and treatment when inappropriately delayed.^21^

Isolated sphenoid sinus disease is an uncommon disease that affects 1%–2.7% of patients with diagnosis of paranasal sinus disease.^21^, ^22^ However, isolated sphenoid sinus disease can be underreported for a number of reasons.^23^ First of all, the presenting symptoms are often nonspecific; secondly the inaccessibility of the sinus precludes optimal physical examination.^23^ Before the advent of CT and MRI scanning, diagnosis of these pathologies was inadequate.^23^

Various pathological findings in the region, including vascular neoplasm, vascular malformations, and encephaloceles, can increase the risk of surgery in this region.^6^ The differential diagnosis includes antrochoanal polyp, hypertrophied adenoids, Thornwaldt cyst pituitary tumours, lymphoma, fungal disease, inverted papillomas, foreign body and malignant tumors.^24^ In the major published series, inflammatory etiologies were responsible for 61%–82% of isolated sphenoid lesions.^21^

Space-occupying lesions such as tumors and mucoceles are more likely to present with visual changes than with inflammatory disease.^23^

Radiological imaging with CT scanning of paranasal sinuses is regarded as the gold standard in the diagnosis of isolated sphenoid sinus disease.^25^ CT is useful in determining the extend of the lesion and identifying focal dehiscence’s within the sinus walls.^25^ MRI is an essential adjunct in the diagnosis and treatment of lesions of the sphenoid sinus. When the diagnosis is difficult, or another pathology is considered, an MRI can be requested as a supplementary imaging modality. As the sphenochoanal polyps may reach huge dimensions, it may be difficult to differentiate it from rhabdomyosarcoma, malignant melanoma, angiofibroma, inverted papilloma and other pedunculated tumors.^7^ Misdiagnosis may cause abundant bleeding or inadequate therapy during surgery.^7^

Partial or complete opacification may suggest lesions such as sinusitis which do not necessitate an MRI.^25^ A globular mass filling the sinus usually represents a mucous retention cyst or polyp.^25^ When thinning, expansion or remodeling of a sinus wall is noted MRI helps to differentiate mucocele from benign tumors.^25^ Evidence of bone erosion and perisinus extension are hallmarks of malignant disease, including that primary in the sinus and those extending from adjacent areas.^25^ MRI is more sensitive than CT scan in identifying fungal concretions within the sphenoid sinus.^21^

In study by Nour et al. MRI was performed in patients with isolated sphenoid sinus pathology: in patients with CSF rhinorrhea to exclude the encephaloceles; in lesions with evident intracranial extension to determine the interface with the neighbouring structures; in lesions with hyperattenuated intrasinus signals to exclude fibroosseous conditions or rare osteoid/chondroid matrix-producing sinonasal sarcomas or meningiomas, and in patients presenting with visual disturbance to rule out any concomitant intracranial pathology.^21^

The average age of children presenting with sphenochoanal polyps was 12 years. As noted by Clement et al. an understanding the development of the sphenoid sinus is helpful in understanding the age of presentation for these children.^17^ The sphenoid sinus is present in a rudimentary form in some neonates, present in 50%–60% of 2-year-olds and in the majority of 6–8-year-olds with a rapid growth phase occurring between the ages of 3–10-years.^17^

Until the 1980s, the most widely used sphenoid approach was an external approach, through a Lynch incision and ethmoidectomy.^21^ The application of the rigid nasal endoscopes and mucosa preserving “functional” principles modernized the treatment of paranasal sinus diseases.^21^ Nowadays, endoscopic sinus surgery is an effective treatment of uncommon choanal polyps.^15^ It is now considered the “gold standard” — the first choice for primary treatment in children. Simple polypectomy or polyp avulsion carries a higher risk of recurrence.^3^ When the polyp is not completely removed, the part left within the sphenoid sinus can cause recurrence.^1^, ^26^ The polyp’s site of origin should be removed to prevent recurrences. This is universal rule guiding the removal of all types of choanal polyps.

The review shows, that 1 recurrence was noted in patient with sphenochoanal polyp, who was primarily diagnosed with antrochoanal polyp and underwent inadequate treatment not involving sphenoidotomy.^14^ The recurrence was noted 4-months after the first surgery.^14^ The authors noted that the major factors leading to an incorrect diagnosis of antrochoanal polyp when a sphenochoanal polyp is present are: the ignorance of the sphenoid sinus pathology; and sphenochoanal polyp causing obstruction of the maxillary sinus, obstruction, which could wrongly lead to the diagnosis of antrochoanal polyp.^14^ An incorrect diagnosis leads to an inappropriate surgical procedure with no exploration of the sphenoid sinus, and sometimes not necessary antrostomy which could have been avoided.^14^ Surgical control of sphenoid sinus must be complete.^14^ In difficult to access pathology, the use of laser for total eradication of the disease can be beneficial.

Some authors reported spontaneous regression of small sphenochoanal polyps. Lim et al. described the regression of a sphenochoanal polyp in a 3-year-old child.^13^ The child underwent adenoidectomy, during which the left choanal mass approximately 1.5 cm in diameter was diagnosed.^13^ Postoperatively, the child was asymptomatic. Although her symptoms resolved, CT scan was performed, revealing an opaque left sphenoid sinus consistent with a polyp, which sat in the left posterior choana.^13^ Endoscopic sinus surgery was undertaken 5-months after adenotonsillectomy, during the surgery no pathology within sphenoid sinus was found.^13^

## Conclusions

A review showed that only 11 cases of sphenochoanal polyps in children have been described in the world literature in English.

Sphenochoanal polyps should be kept in mind in the differential diagnosis of unilateral nasal cavity or paranasal sinuses masses. Misdiagnosis can result in recurrences in patients with sphenochoanal polyp, who can be mistakenly diagnosed with antrochoanal polyp and underwent inadequate treatment not involving sphenoidotomy and exact identification of the site of implantation.

The symptoms of sphenochoanal polyps are nonspecific. Children most commonly present with nasal obstruction and discharge. Radiological imaging with CT scanning of paranasal sinuses is regarded as the gold standard in the diagnosis of isolated sphenoid sinus disease. When sphenoid opacification is present total eradication of the disease is mandatory. When the diagnosis is difficult or another pathology is considered, an MRI can be requested as a supplementary imaging modality. The endoscopic sinus surgery is now considered the “gold standard” — the first choice for primary treatment in children.

## Funding

This research did not receive any specific grant from funding agencies in the public, commercial, or not-for-profit sectors.

## Ethical approval

Not applicable – systematic review – ethical approval not required for the study.

## Conflicts of interest

The authors declare no conflicts of interest.

## References

[1] Çeçen A., Kemal O., Atmaca S., Kavaz E. (2017). Isolated sphenochoanal polyp: report of three cases. Hippokratia.

[2] Kumral T.L., Yildirim G., Uyar Y. (2012). Sphenochoanal polyps and the optic nerve. Clin Pract.

[3] Özdek A., Erdem H. (2014). Unusual presentations of choanal polyps: report of 3 cases. Ear Nose Throat J.

[4] Mantilla E., Villamor P., De La Torre C., Álvarez-Neri H. (2019). Combined approach for paediatric recurrent antrochoanal polyp: a single-centre case series of 27 children. J Laryngol Otol.

[5] Yuca K., Bayram Ï., Kiroǧlu A.F., Etlik O., Cankaya H., Sakin F. (2006). Evaluation and treatment of antrochoanal polyps. J Otolaryngol.

[6] Jadia S., Goyal R., Biswas R. (2010). Nasal mass mimicking antrochoanal polyp. BMJ Case Rep.

[7] Tosun F., Yetiser S., Akcam T., Özkaptan Y. (2001). Sphenochoanal polyp: endoscopic surgery. Int J Pediatr Otorhinolaryngol.

[8] Gursoy M., Erdogan N., Cetinoglu Y., Dag F., Eren E., Uluc M. (2019). Anatomic variations associated with antrochoanal polyps. Niger J Clin Pract.

[9] Özdek A., Samim E., Bayiz Ü., Meral I., Şafak M.A., Oǧuz H. (2002). Antrochoanal polyps in children. Int J Pediatr Otorhinolaryngol.

[10] Galluzzi F., Pignataro L., Maddalone M., Garavello W. (2018). Recurrences of surgery for antrochoanal polyps in children: a systematic review. Int J Pediatr Otorhinolaryngol.

[11] Moher D., Altman D.G., Liberati A., Tetzlaff J. (2011). PRISMA statement. Epidemiology.

[12] Al-Qudah M.A. (2010). Sphenochoanal polyp: current diagnosis and management. Ear Nose Throat J.

[13] Lim W.K., Sdralis T. (2004). Regression of a sphenochoanal polyp in a child. Laryngoscope.

[14] Crampette L., Mondain M., Rombaux P. (1995). Sphenochoanal polyp in children. Diagnosis and treatment. Rhinology.

[15] Íleri F., Köybaşioǧlu A., Uslu S. (1998). Clinical presentation of a sphenochoanal polyp. Eur Arch Otorhinolaryngol.

[16] Yanagisawa K., Steven H., Yanagisawa E. (2000). Endoscopic view of a sphenochoanal polyp. Ear Nose Throat J.

[17] Clement W.A., Sooby P., Doherty C., Qayyum N., Irwin G. (2021). Acute isolated sphenoid sinusitis in children: a case series and systematic review of the literature. Int J Pediatr Otorhinolaryngol.

[18] De Vuysere S., Hermans R., Marchal G. (2001). Sinochoanal polyp and its variant, the angiomatous polyp: MRI findings. Eur Radiol.

[19] Spraggs P.D.R. (1993). Radiological diagnosis of spheno-choanal polyp. J Laryngol Otol.

[20] Veloso I., Nunes C., Rimoli C., Tagliarini J., Melo L., Queiroga T. (2014). Sphenocoanal polyp in an 8-year-old child. Int Arch Otorhinolaryngol.

[21] Nour Y.A., Al-Madani A., El-Daly A., Gaafar A. (2008). Isolated sphenoid sinus pathology: spectrum of diagnostic and treatment modalities. Auris Nasus Larynx.

[22] Polak M., Mierzwiński J., Lasek W., Piziewicz A., Muller M. (2000). Izolowane zapalenie zatoki klinowej u dziecka leczone metoda endoskopowa. Polish Otolaryngol J.

[23] Sethi D.S. (1999). Isolated sphenoid lesions: diagnosis and management. Otolaryngol Head Neck Surg.

[24] Wang Z.M., Kanoh N., Dai C.F., Kutler D.I., Xu R., Chi F.L. (2002). Isolated sphenoid sinus disease: an analysis of 122 cases. Ann Otol Rhinol Laryngol.

[25] Manjula B.V., Nair A.B., Balasubramanyam A.M., Tandon S., Nayar R.C. (2010). Isolated sphenoid sinus disease — a retrospective analysis. Indian J Otolaryngol Head Neck Surg.

[26] Bist S.S., Kumar R., Varshney S., Bisht M. (2007). Isolated sphenochoanal polyp: a rare clinical entity. Indian J Otolaryngol Head Neck Surg.

